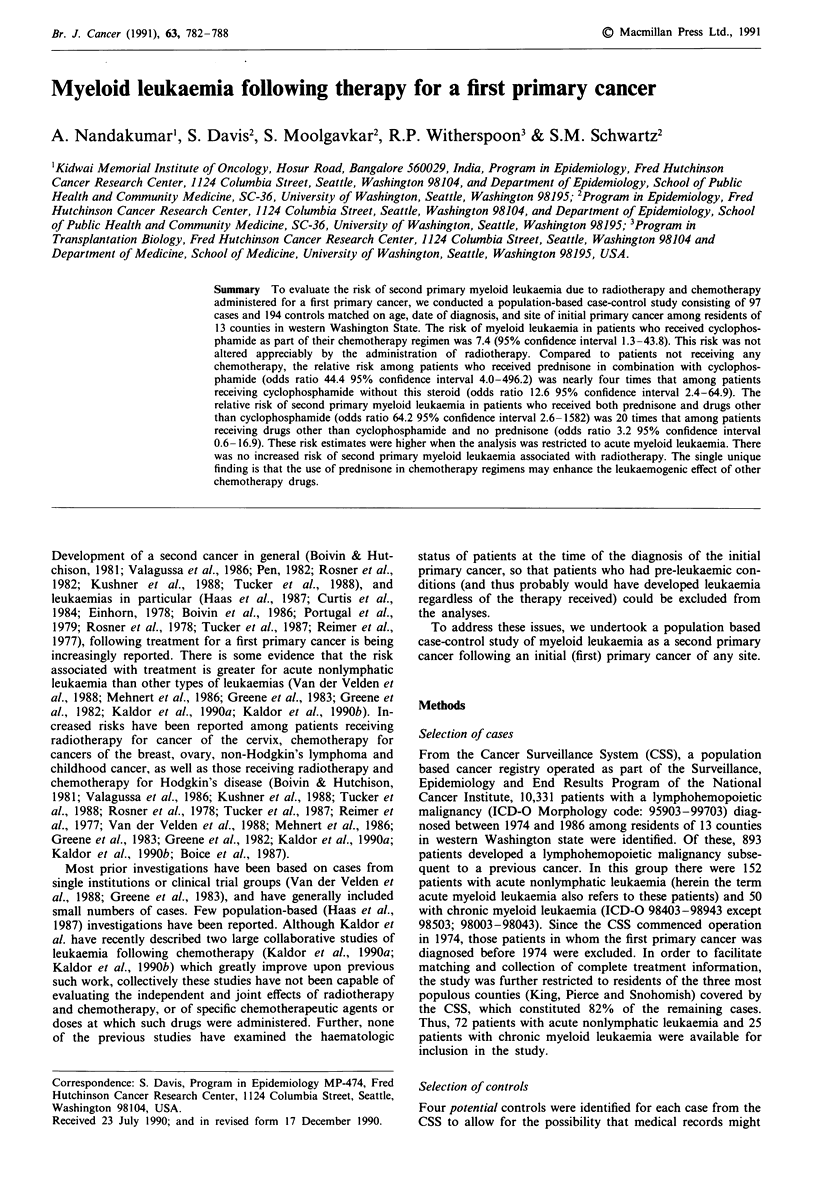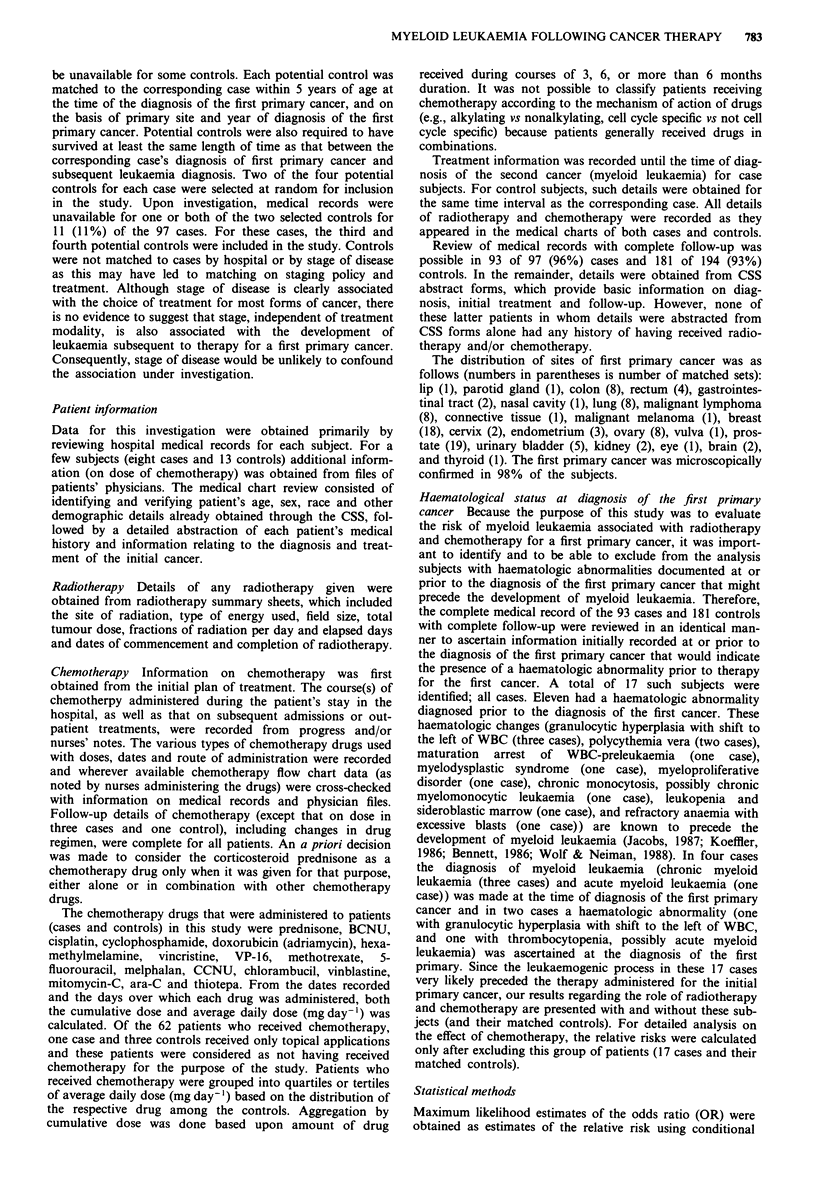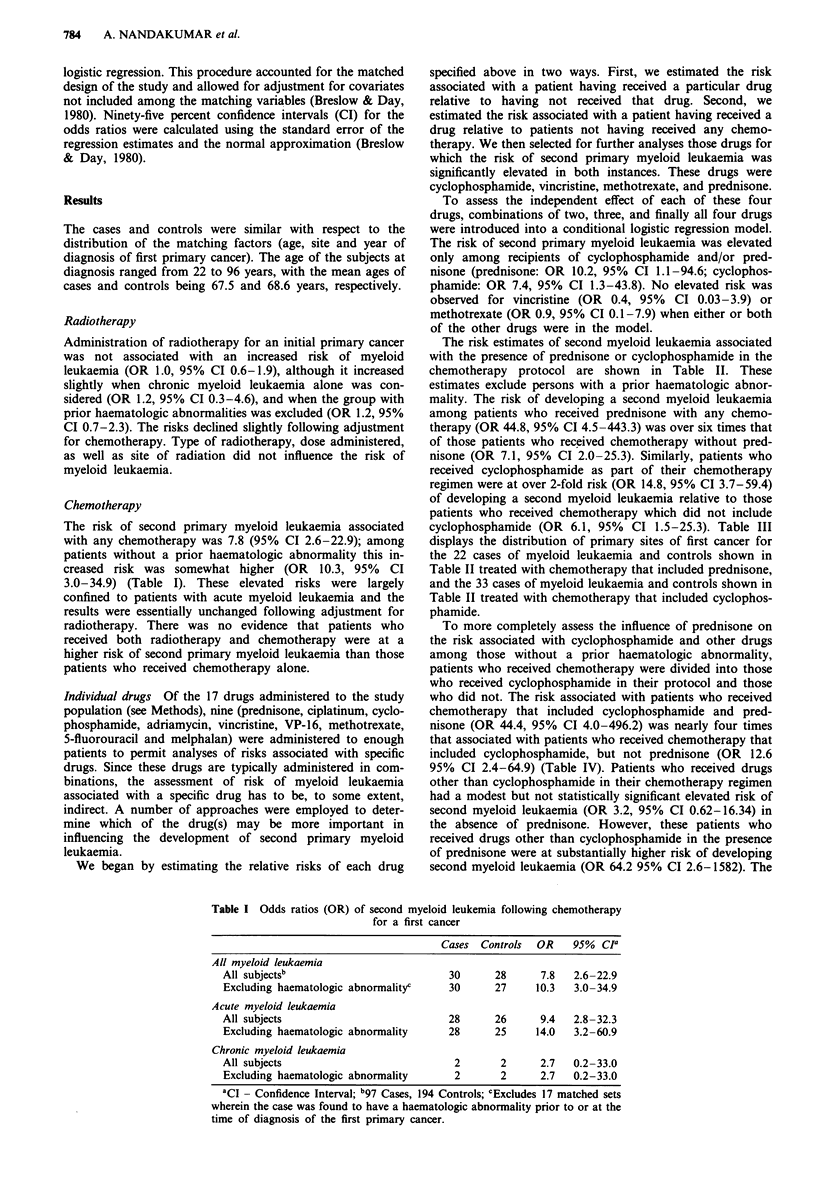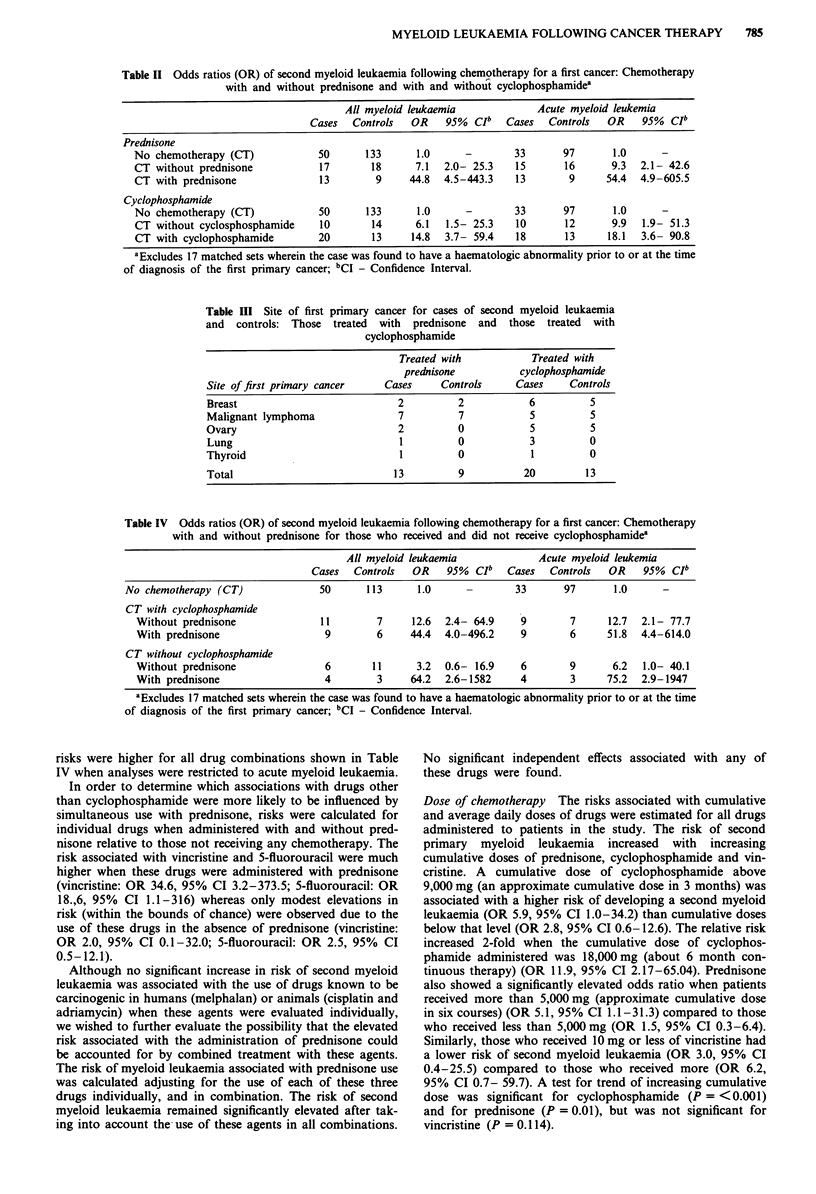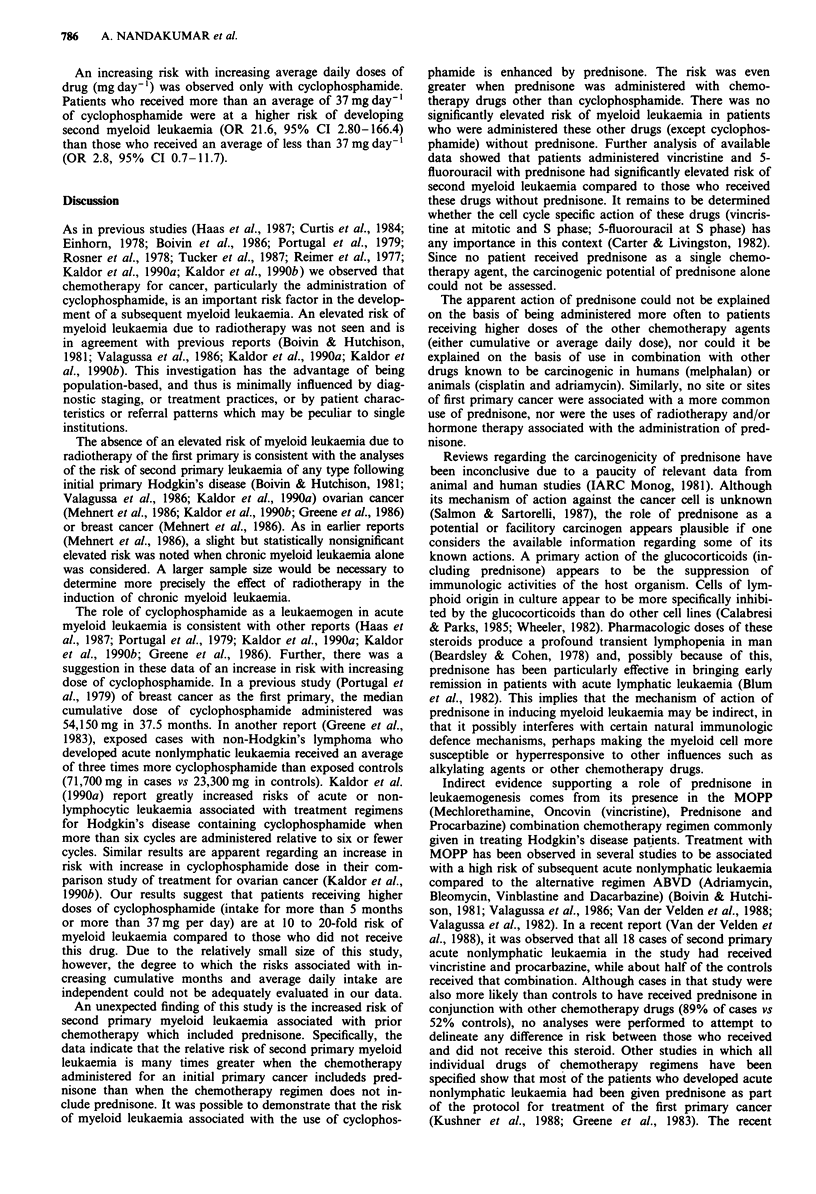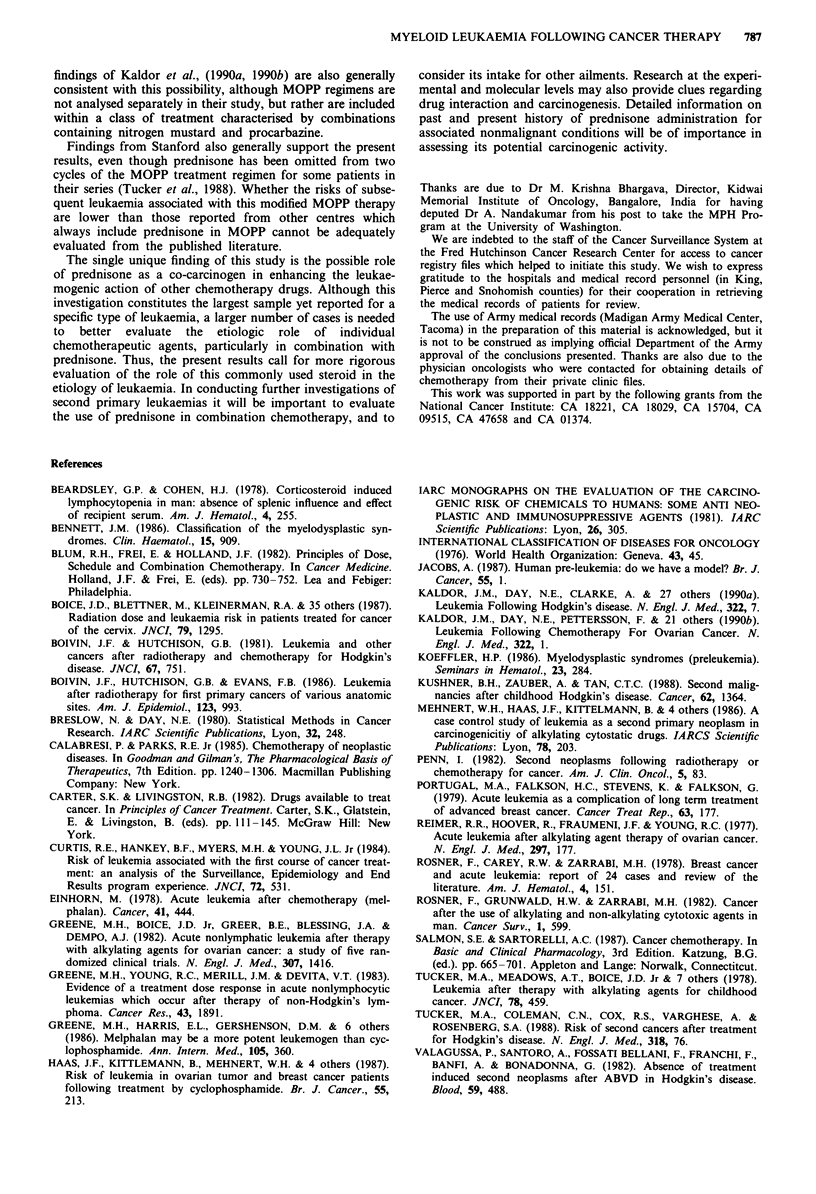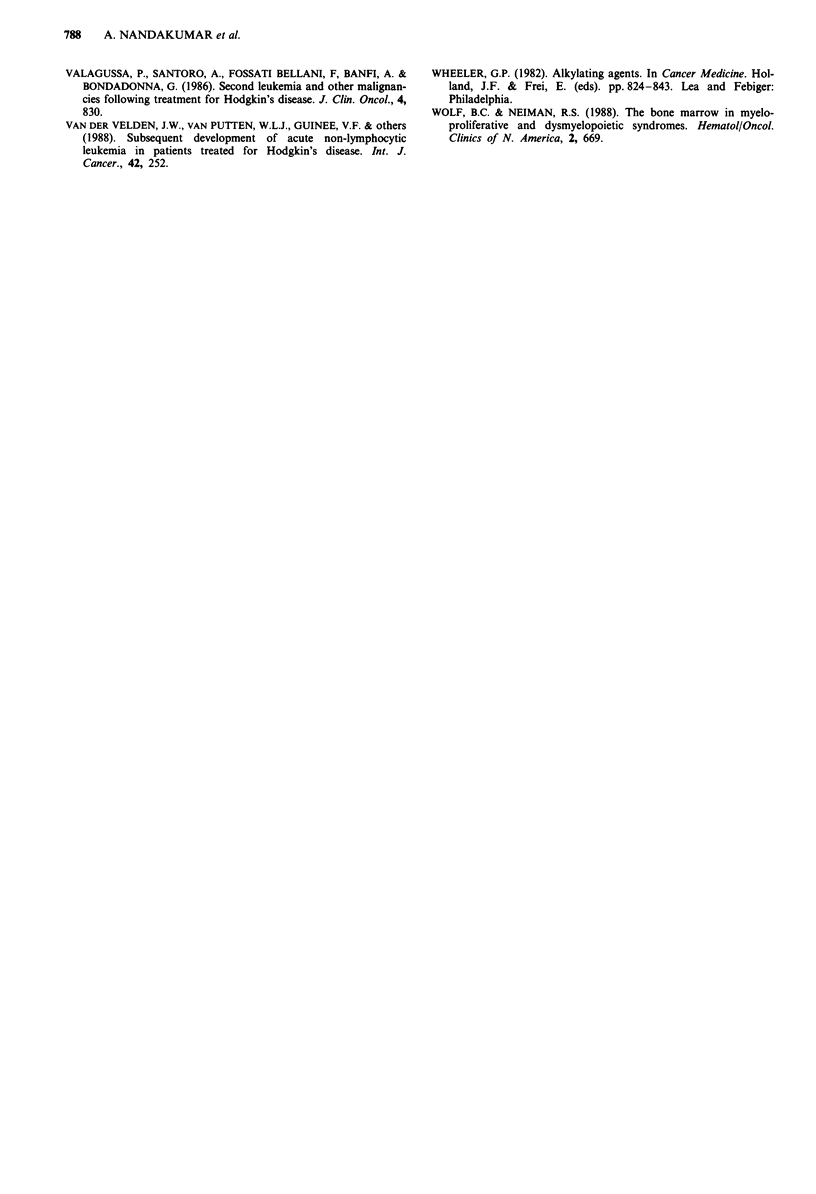# Myeloid leukaemia following therapy for a first primary cancer.

**DOI:** 10.1038/bjc.1991.174

**Published:** 1991-05

**Authors:** A. Nandakumar, S. Davis, S. Moolgavkar, R. P. Witherspoon, S. M. Schwartz

**Affiliations:** Kidwai Memorial Institute of Oncology, Bangalore, India.

## Abstract

To evaluate the risk of second primary myeloid leukaemia due to radiotherapy and chemotherapy administered for a first primary cancer, we conducted a population-based case-control study consisting of 97 cases and 194 controls matched on age, date of diagnosis, and site of initial primary cancer among residents of 13 counties in western Washington State. The risk of myeloid leukaemia in patients who received cyclophosphamide as part of their chemotherapy regimen was 7.4 (95% confidence interval 1.3-43.8). This risk was not altered appreciably by the administration of radiotherapy. Compared to patients not receiving any chemotherapy, the relative risk among patients who received prednisone in combination with cyclophosphamide (odds ratio 44.4 95% confidence interval 4.0-496.2) was nearly four times that among patients receiving cyclophosphamide without this steroid (odds ratio 12.6 95% confidence interval 2.4-64.9). The relative risk of second primary myeloid leukaemia in patients who received both prednisone and drugs other than cyclophosphamide (odds ratio 64.2 95% confidence interval 2.6-1582) was 20 times that among patients receiving drugs other than cyclophosphamide and no prednisone (odds ratio 3.2 95% confidence interval 0.6-16.9). These risk estimates were higher when the analysis was restricted to acute myeloid leukaemia. There was no increased risk of second primary myeloid leukaemia associated with radiotherapy. The single unique finding is that the use of prednisone in chemotherapy regimens may enhance the leukaemogenic effect of other chemotherapy drugs.


Br. J. Cancer (1991), 63, 782–788 © Macmillan Press Ltd., 1991

### Myeloid leukaemia following therapy for a first primary cancer

A. Nandakumar[1], S. Davis[2], S. Moolgavkar[2], R.P. Witherspoon[3] & S.M. Schwartz[2]

[1]*Kidwai Memorial Institute of Oncology, Hosur Road, Bangalore 560029, India, Program in Epidemiology, Fred Hutchinson Cancer Research Center, 1124 Columbia Street, Seattle, Washington 98104, and Department of Epidemiology, School of Public Health and Community Medicine, SC-36, University of Washington, Seattle, Washington 98195;* [2]*Program in Epidemiology, Fred Hutchinson Cancer Research Center, 1124 Columbia Street, Seattle, Washington 98104, and Department of Epidemiology, School of Public Health and Community Medicine, SC-36, University of Washington, Seattle, Washington 98195;* [3]*Program in Transplantation Biology, Fred Hutchinson Cancer Research Center, 1124 Columbia Street, Seattle, Washington 98104 and Department of Medicine, School of Medicine, University of Washington, Seattle, Washington 98195, USA.*

**Summary** To evaluate the risk of second primary myeloid leukaemia due to radiotherapy and chemotherapy administered for a first primary cancer, we conducted a population-based case-control study consisting of 97 cases and 194 controls matched on age, date of diagnosis, and site of initial primary cancer among residents of 13 counties in western Washington State. The risk of myeloid leukaemia in patients who received cyclophosphamide as part of their chemotherapy regimen was 7.4 (95% confidence interval 1.3–43.8). This risk was not altered appreciably by the administration of radiotherapy. Compared to patients not receiving any chemotherapy, the relative risk among patients who received prednisone in combination with cyclophosphamide (odds ratio 44.4 95% confidence interval 4.0–496.2) was nearly four times that among patients receiving cyclophosphamide without this steroid (odds ratio 12.6 95% confidence interval 2.4–64.9). The relative risk of second primary myeloid leukaemia in patients who received both prednisone and drugs other than cyclophosphamide (odds ratio 64.2 95% confidence interval 2.6–1582) was 20 times that among patients receiving drugs other than cyclophosphamide and no prednisone (odds ratio 3.2 95% confidence interval 0.6–16.9). These risk estimates were higher when the analysis was restricted to acute myeloid leukaemia. There was no increased risk of second primary myeloid leukaemia associated with radiotherapy. The single unique finding is that the use of prednisone in chemotherapy regimens may enhance the leukaemogenic effect of other chemotherapy drugs.

Development of a second cancer in general (Boivin & Hutchison, 1981; Valagussa *et al.*, 1986; Pen, 1982; Rosner *et al.*, 1982; Kushner *et al.*, 1988; Tucker *et al.*, 1988), and leukaemias in particular (Haas *et al.*, 1987; Curtis *et al.*, 1984; Einhorn, 1978; Boivin *et al.*, 1986; Portugal *et al.*, 1979; Rosner *et al.*, 1978; Tucker *et al.*, 1987; Reimer *et al.*, 1977), following treatment for a first primary cancer is being increasingly reported. There is some evidence that the risk associated with treatment is greater for acute nonlymphatic leukaemia than other types of leukaemias (Van der Velden *et al.*, 1988; Mehnert *et al.*, 1986; Greene *et al.*, 1983; Greene *et al.*, 1982; Kaldor *et al.*, 1990*a*; Kaldor *et al.*, 1990*b*). Increased risks have been reported among patients receiving radiotherapy for cancer of the cervix, chemotherapy for cancers of the breast, ovary, non-Hodgkin's lymphoma and childhood cancer, as well as those receiving radiotherapy and chemotherapy for Hodgkin's disease (Boivin & Hutchison, 1981; Valagussa *et al.*, 1986; Kushner *et al.*, 1988; Tucker *et al.*, 1988; Rosner *et al.*, 1978; Tucker *et al.*, 1987; Reimer *et al.*, 1977; Van der Velden *et al.*, 1988; Mehnert *et al.*, 1986; Greene *et al.*, 1983; Greene *et al.*, 1982; Kaldor *et al.*, 1990*a*; Kaldor *et al.*, 1990*b*; Boice *et al.*, 1987).

Most prior investigations have been based on cases from single institutions or clinical trial groups (Van der Velden *et al.*, 1988; Greene *et al.*, 1983), and have generally included small numbers of cases. Few population-based (Haas *et al.*, 1987) investigations have been reported. Although Kaldor *et al.* have recently described two large collaborative studies of leukaemia following chemotherapy (Kaldor *et al.*, 1990*a*; Kaldor *et al.*, 1990*b*) which greatly improve upon previous such work, collectively these studies have not been capable of evaluating the independent and joint effects of radiotherapy and chemotherapy, or of specific chemotherapeutic agents or doses at which such drugs were administered. Further, none of the previous studies have examined the haematologic status of patients at the time of the diagnosis of the initial primary cancer, so that patients who had pre-leukaemic conditions (and thus probably would have developed leukaemia regardless of the therapy received) could be excluded from the analyses.

To address these issues, we undertook a population based case-control study of myeloid leukaemia as a second primary cancer following an initial (first) primary cancer of any site.

### Methods

### *Selection of cases*

From the Cancer Surveillance System (CSS), a population based cancer registry operated as part of the Surveillance, Epidemiology and End Results Program of the National Cancer Institute, 10,331 patients with a lymphohemopoietic malignancy (ICD-O Morphology code: 95903–99703) diagnosed between 1974 and 1986 among residents of 13 counties in western Washington state were identified. Of these, 893 patients developed a lymphohemopoietic malignancy subsequent to a previous cancer. In this group there were 152 patients with acute nonlymphatic leukaemia (herein the term acute myeloid leukaemia also refers to these patients) and 50 with chronic myeloid leukaemia (ICD-O 98403–98943 except 98503; 98003–98043). Since the CSS commenced operation in 1974, those patients in whom the first primary cancer was diagnosed before 1974 were excluded. In order to facilitate matching and collection of complete treatment information, the study was further restricted to residents of the three most populous counties (King, Pierce and Snohomish) covered by the CSS, which constituted 82% of the remaining cases. Thus, 72 patients with acute nonlymphatic leukaemia and 25 patients with chronic myeloid leukaemia were available for inclusion in the study.

### *Selection of controls*

Four *potential* controls were identified for each case from the CSS to allow for the possibility that medical records might

Correspondence: S. Davis, Program in Epidemiology MP-474, Fred Hutchinson Cancer Research Center, 1124 Columbia Street, Seattle, Washington 98104, USA.
Received 23 July 1990; and in revised form 17 December 1990.

be unavailable for some controls. Each potential control was matched to the corresponding case within 5 years of age at the time of the diagnosis of the first primary cancer, and on the basis of primary site and year of diagnosis of the first primary cancer. Potential controls were also required to have survived at least the same length of time as that between the corresponding case's diagnosis of first primary cancer and subsequent leukaemia diagnosis. Two of the four potential controls for each case were selected at random for inclusion in the study. Upon investigation, medical records were unavailable for one or both of the two selected controls for 11 (11%) of the 97 cases. For these cases, the third and fourth potential controls were included in the study. Controls were not matched to cases by hospital or by stage of disease as this may have led to matching on staging policy and treatment. Although stage of disease is clearly associated with the choice of treatment for most forms of cancer, there is no evidence to suggest that stage, independent of treatment modality, is also associated with the development of leukaemia subsequent to therapy for a first primary cancer. Consequently, stage of disease would be unlikely to confound the association under investigation.

*Patient information*

Data for this investigation were obtained primarily by reviewing hospital medical records for each subject. For a few subjects (eight cases and 13 controls) additional information (on dose of chemotherapy) was obtained from files of patients' physicians. The medical chart review consisted of identifying and verifying patient's age, sex, race and other demographic details already obtained through the CSS, followed by a detailed abstraction of each patient's medical history and information relating to the diagnosis and treatment of the initial cancer.

*Radiotherapy* Details of any radiotherapy given were obtained from radiotherapy summary sheets, which included the site of radiation, type of energy used, field size, total tumour dose, fractions of radiation per day and elapsed days and dates of commencement and completion of radiotherapy.

*Chemotherapy* Information on chemotherapy was first obtained from the initial plan of treatment. The course(s) of chemotherpy administered during the patient's stay in the hospital, as well as that on subsequent admissions or outpatient treatments, were recorded from progress and/or nurses' notes. The various types of chemotherapy drugs used with doses, dates and route of administration were recorded and wherever available chemotherapy flow chart data (as noted by nurses administering the drugs) were cross-checked with information on medical records and physician files. Follow-up details of chemotherapy (except that on dose in three cases and one control), including changes in drug regimen, were complete for all patients. An *a priori* decision was made to consider the corticosteroid prednisone as a chemotherapy drug only when it was given for that purpose, either alone or in combination with other chemotherapy drugs.

The chemotherapy drugs that were administered to patients (cases and controls) in this study were prednisone, BCNU, cisplatin, cyclophosphamide, doxorubicin (adriamycin), hexamethylmelamine, vincristine, VP-16, methotrexate, 5-fluorouracil, melphalan, CCNU, chlorambucil, vinblastine, mitomycin-C, ara-C and thiotepa. From the dates recorded and the days over which each drug was administered, both the cumulative dose and average daily dose (mg $day^{-1}$) was calculated. Of the 62 patients who received chemotherapy, one case and three controls received only topical applications and these patients were considered as not having received chemotherapy for the purpose of the study. Patients who received chemotherapy were grouped into quartiles or tertiles of average daily dose (mg $day^{-1}$) based on the distribution of the respective drug among the controls. Aggregation by cumulative dose was done based upon amount of drug received during courses of 3, 6, or more than 6 months duration. It was not possible to classify patients receiving chemotherapy according to the mechanism of action of drugs (e.g., alkylating *vs* nonalkylating, cell cycle specific *vs* not cell cycle specific) because patients generally received drugs in combinations.

Treatment information was recorded until the time of diagnosis of the second cancer (myeloid leukaemia) for case subjects. For control subjects, such details were obtained for the same time interval as the corresponding case. All details of radiotherapy and chemotherapy were recorded as they appeared in the medical charts of both cases and controls.

Review of medical records with complete follow-up was possible in 93 of 97 (96%) cases and 181 of 194 (93%) controls. In the remainder, details were obtained from CSS abstract forms, which provide basic information on diagnosis, initial treatment and follow-up. However, none of these latter patients in whom details were abstracted from CSS forms alone had any history of having received radiotherapy and/or chemotherapy.

The distribution of sites of first primary cancer was as follows (numbers in parentheses is number of matched sets): lip (1), parotid gland (1), colon (8), rectum (4), gastrointestinal tract (2), nasal cavity (1), lung (8), malignant lymphoma (8), connective tissue (1), malignant melanoma (1), breast (18), cervix (2), endometrium (3), ovary (8), vulva (1), prostate (19), urinary bladder (5), kidney (2), eye (1), brain (2), and thyroid (1). The first primary cancer was microscopically confirmed in 98% of the subjects.

*Haematological status at diagnosis of the first primary cancer* Because the purpose of this study was to evaluate the risk of myeloid leukaemia associated with radiotherapy and chemotherapy for a first primary cancer, it was important to identify and to be able to exclude from the analysis subjects with haematologic abnormalities documented at or prior to the diagnosis of the first primary cancer that might precede the development of myeloid leukaemia. Therefore, the complete medical record of the 93 cases and 181 controls with complete follow-up were reviewed in an identical manner to ascertain information initially recorded at or prior to the diagnosis of the first primary cancer that would indicate the presence of a haematologic abnormality prior to therapy for the first cancer. A total of 17 such subjects were identified; all cases. Eleven had a haematologic abnormality diagnosed prior to the diagnosis of the first cancer. These haematologic changes (granulocytic hyperplasia with shift to the left of WBC (three cases), polycythemia vera (two cases), maturation arrest of WBC-preleukaemia (one case), myelodysplastic syndrome (one case), myeloproliferative disorder (one case), chronic monocytosis, possibly chronic myelomonocytic leukaemia (one case), leukopenia and sideroblastic marrow (one case), and refractory anaemia with excessive blasts (one case)) are known to precede the development of myeloid leukaemia (Jacobs, 1987; Koeffler, 1986; Bennett, 1986; Wolf & Neiman, 1988). In four cases the diagnosis of myeloid leukaemia (chronic myeloid leukaemia (three cases) and acute myeloid leukaemia (one case)) was made at the time of diagnosis of the first primary cancer and in two cases a haematologic abnormality (one with granulocytic hyperplasia with shift to the left of WBC, and one with thrombocytopenia, possibly acute myeloid leukaemia) was ascertained at the diagnosis of the first primary. Since the leukaemogenic process in these 17 cases very likely preceded the therapy administered for the initial primary cancer, our results regarding the role of radiotherapy and chemotherapy are presented with and without these subjects (and their matched controls). For detailed analysis on the effect of chemotherapy, the relative risks were calculated only after excluding this group of patients (17 cases and their matched controls).

*Statistical methods*

Maximum likelihood estimates of the odds ratio (OR) were obtained as estimates of the relative risk using conditional logistic regression. This procedure accounted for the matched design of the study and allowed for adjustment for covariates not included among the matching variables (Breslow & Day, 1980). Ninety-five percent confidence intervals (CI) for the odds ratios were calculated using the standard error of the regression estimates and the normal approximation (Breslow & Day, 1980).

### Results

The cases and controls were similar with respect to the distribution of the matching factors (age, site and year of diagnosis of first primary cancer). The age of the subjects at diagnosis ranged from 22 to 96 years, with the mean ages of cases and controls being 67.5 and 68.6 years, respectively.

### *Radiotherapy*

Administration of radiotherapy for an initial primary cancer was not associated with an increased risk of myeloid leukaemia (OR 1.0, 95% CI 0.6–1.9), although it increased slightly when chronic myeloid leukaemia alone was considered (OR 1.2, 95% CI 0.3–4.6), and when the group with prior haematologic abnormalities was excluded (OR 1.2, 95% CI 0.7–2.3). The risks declined slightly following adjustment for chemotherapy. Type of radiotherapy, dose administered, as well as site of radiation did not influence the risk of myeloid leukaemia.

### *Chemotherapy*

The risk of second primary myeloid leukaemia associated with any chemotherapy was 7.8 (95% CI 2.6–22.9); among patients without a prior haematologic abnormality this increased risk was somewhat higher (OR 10.3, 95% CI 3.0–34.9) (Table I). These elevated risks were largely confined to patients with acute myeloid leukaemia and the results were essentially unchanged following adjustment for radiotherapy. There was no evidence that patients who received both radiotherapy and chemotherapy were at a higher risk of second primary myeloid leukaemia than those patients who received chemotherapy alone.

*Individual drugs* Of the 17 drugs administered to the study population (see Methods), nine (prednisone, ciplatinum, cyclophosphamide, adriamycin, vincristine, VP-16, methotrexate, 5-fluorouracil and melphalan) were administered to enough patients to permit analyses of risks associated with specific drugs. Since these drugs are typically administered in combinations, the assessment of risk of myeloid leukaemia associated with a specific drug has to be, to some extent, indirect. A number of approaches were employed to determine which of the drug(s) may be more important in influencing the development of second primary myeloid leukaemia.

We began by estimating the relative risks of each drug specified above in two ways. First, we estimated the risk associated with a patient having received a particular drug relative to having not received that drug. Second, we estimated the risk associated with a patient having received a drug relative to patients not having received any chemotherapy. We then selected for further analyses those drugs for which the risk of second primary myeloid leukaemia was significantly elevated in both instances. These drugs were cyclophosphamide, vincristine, methotrexate, and prednisone.

To assess the independent effect of each of these four drugs, combinations of two, three, and finally all four drugs were introduced into a conditional logistic regression model. The risk of second primary myeloid leukaemia was elevated only among recipients of cyclophosphamide and/or prednisone (prednisone: OR 10.2, 95% CI 1.1–94.6; cyclophosphamide: OR 7.4, 95% CI 1.3–43.8). No elevated risk was observed for vincristine (OR 0.4, 95% CI 0.03–3.9) or methotrexate (OR 0.9, 95% CI 0.1–7.9) when either or both of the other drugs were in the model.

The risk estimates of second myeloid leukaemia associated with the presence of prednisone or cyclophosphamide in the chemotherapy protocol are shown in Table II. These estimates exclude persons with a prior haematologic abnormality. The risk of developing a second myeloid leukaemia among patients who received prednisone with any chemotherapy (OR 44.8, 95% CI 4.5–443.3) was over six times that of those patients who received chemotherapy without prednisone (OR 7.1, 95% CI 2.0–25.3). Similarly, patients who received cyclophosphamide as part of their chemotherapy regimen were at over 2-fold risk (OR 14.8, 95% CI 3.7–59.4) of developing a second myeloid leukaemia relative to those patients who received chemotherapy which did not include cyclophosphamide (OR 6.1, 95% CI 1.5–25.3). Table III displays the distribution of primary sites of first cancer for the 22 cases of myeloid leukaemia and controls shown in Table II treated with chemotherapy that included prednisone, and the 33 cases of myeloid leukaemia and controls shown in Table II treated with chemotherapy that included cyclophosphamide.

To more completely assess the influence of prednisone on the risk associated with cyclophosphamide and other drugs among those without a prior haematologic abnormality, patients who received chemotherapy were divided into those who received cyclophosphamide in their protocol and those who did not. The risk associated with patients who received chemotherapy that included cyclophosphamide and prednisone (OR 44.4, 95% CI 4.0–496.2) was nearly four times that associated with patients who received chemotherapy that included cyclophosphamide, but not prednisone (OR 12.6 95% CI 2.4–64.9) (Table IV). Patients who received drugs other than cyclophosphamide in their chemotherapy regimen had a modest but not statistically significant elevated risk of second myeloid leukaemia (OR 3.2, 95% CI 0.62–16.34) in the absence of prednisone. However, these patients who received drugs other than cyclophosphamide in the presence of prednisone were at substantially higher risk of developing second myeloid leukaemia (OR 64.2 95% CI 2.6–1582). The

**Table I** Odds ratios (OR) of second myeloid leukemia following chemotherapy for a first cancer

| | *Cases* | *Controls* | *OR* | *95% CI*[a] |
|---|---|---|---|---|
| *All myeloid leukaemia* | | | | |
| All subjects[b] | 30 | 28 | 7.8 | 2.6–22.9 |
| Excluding haematologic abnormality[c] | 30 | 27 | 10.3 | 3.0–34.9 |
| *Acute myeloid leukaemia* | | | | |
| All subjects | 28 | 26 | 9.4 | 2.8–32.3 |
| Excluding haematologic abnormality | 28 | 25 | 14.0 | 3.2–60.9 |
| *Chronic myeloid leukaemia* | | | | |
| All subjects | 2 | 2 | 2.7 | 0.2–33.0 |
| Excluding haematologic abnormality | 2 | 2 | 2.7 | 0.2–33.0 |

[a]CI – Confidence Interval; [b]97 Cases, 194 Controls; [c]Excludes 17 matched sets wherein the case was found to have a haematologic abnormality prior to or at the time of diagnosis of the first primary cancer.

**Table II** Odds ratios (OR) of second myeloid leukaemia following chemotherapy for a first cancer: Chemotherapy with and without prednisone and with and without cyclophosphamide[a]

| | All myeloid leukaemia | | | | Acute myeloid leukemia | | | |
|---|---|---|---|---|---|---|---|---|
| | *Cases* | *Controls* | *OR* | *95% CI*[b] | *Cases* | *Controls* | *OR* | *95% CI*[b] |
| *Prednisone* | | | | | | | | |
| No chemotherapy (CT) | 50 | 133 | 1.0 | – | 33 | 97 | 1.0 | – |
| CT without prednisone | 17 | 18 | 7.1 | 2.0– 25.3 | 15 | 16 | 9.3 | 2.1– 42.6 |
| CT with prednisone | 13 | 9 | 44.8 | 4.5–443.3 | 13 | 9 | 54.4 | 4.9–605.5 |
| *Cyclophosphamide* | | | | | | | | |
| No chemotherapy (CT) | 50 | 133 | 1.0 | – | 33 | 97 | 1.0 | – |
| CT without cyclosphosphamide | 10 | 14 | 6.1 | 1.5– 25.3 | 10 | 12 | 9.9 | 1.9– 51.3 |
| CT with cyclophosphamide | 20 | 13 | 14.8 | 3.7– 59.4 | 18 | 13 | 18.1 | 3.6– 90.8 |

[a]Excludes 17 matched sets wherein the case was found to have a haematologic abnormality prior to or at the time of diagnosis of the first primary cancer; [b]CI – Confidence Interval.

**Table III** Site of first primary cancer for cases of second myeloid leukaemia and controls: Those treated with prednisone and those treated with cyclophosphamide

| | *Treated with prednisone* | | *Treated with cyclophosphamide* | |
|---|---|---|---|---|
| *Site of first primary cancer* | *Cases* | *Controls* | *Cases* | *Controls* |
| Breast | 2 | 2 | 6 | 5 |
| Malignant lymphoma | 7 | 7 | 5 | 5 |
| Ovary | 2 | 0 | 5 | 5 |
| Lung | 1 | 0 | 3 | 0 |
| Thyroid | 1 | 0 | 1 | 0 |
| Total | 13 | 9 | 20 | 13 |

**Table IV** Odds ratios (OR) of second myeloid leukaemia following chemotherapy for a first cancer: Chemotherapy with and without prednisone for those who received and did not receive cyclophosphamide[a]

| | All myeloid leukaemia | | | | Acute myeloid leukemia | | | |
|---|---|---|---|---|---|---|---|---|
| | *Cases* | *Controls* | *OR* | *95% CI*[b] | *Cases* | *Controls* | *OR* | *95% CI*[b] |
| *No chemotherapy (CT)* | 50 | 113 | 1.0 | – | 33 | 97 | 1.0 | – |
| *CT with cyclophosphamide* | | | | | | | | |
| Without prednisone | 11 | 7 | 12.6 | 2.4– 64.9 | 9 | 7 | 12.7 | 2.1– 77.7 |
| With prednisone | 9 | 6 | 44.4 | 4.0–496.2 | 9 | 6 | 51.8 | 4.4–614.0 |
| *CT without cyclophosphamide* | | | | | | | | |
| Without prednisone | 6 | 11 | 3.2 | 0.6– 16.9 | 6 | 9 | 6.2 | 1.0– 40.1 |
| With prednisone | 4 | 3 | 64.2 | 2.6–1582 | 4 | 3 | 75.2 | 2.9–1947 |

[a]Excludes 17 matched sets wherein the case was found to have a haematologic abnormality prior to or at the time of diagnosis of the first primary cancer; [b]CI – Confidence Interval.

risks were higher for all drug combinations shown in Table IV when analyses were restricted to acute myeloid leukaemia.

In order to determine which associations with drugs other than cyclophosphamide were more likely to be influenced by simultaneous use with prednisone, risks were calculated for individual drugs when administered with and without prednisone relative to those not receiving any chemotherapy. The risk associated with vincristine and 5-fluorouracil were much higher when these drugs were administered with prednisone (vincristine: OR 34.6, 95% CI 3.2–373.5; 5-fluorouracil: OR 18.,6, 95% CI 1.1–316) whereas only modest elevations in risk (within the bounds of chance) were observed due to the use of these drugs in the absence of prednisone (vincristine: OR 2.0, 95% CI 0.1–32.0; 5-fluorouracil: OR 2.5, 95% CI 0.5–12.1).

Although no significant increase in risk of second myeloid leukaemia was associated with the use of drugs known to be carcinogenic in humans (melphalan) or animals (cisplatin and adriamycin) when these agents were evaluated individually, we wished to further evaluate the possibility that the elevated risk associated with the administration of prednisone could be accounted for by combined treatment with these agents. The risk of myeloid leukaemia associated with prednisone use was calculated adjusting for the use of each of these three drugs individually, and in combination. The risk of second myeloid leukaemia remained significantly elevated after taking into account the use of these agents in all combinations. No significant independent effects associated with any of these drugs were found.

*Dose of chemotherapy* The risks associated with cumulative and average daily doses of drugs were estimated for all drugs administered to patients in the study. The risk of second primary myeloid leukaemia increased with increasing cumulative doses of prednisone, cyclophosphamide and vincristine. A cumulative dose of cyclophosphamide above 9,000 mg (an approximate cumulative dose in 3 months) was associated with a higher risk of developing a second myeloid leukaemia (OR 5.9, 95% CI 1.0–34.2) than cumulative doses below that level (OR 2.8, 95% CI 0.6–12.6). The relative risk increased 2-fold when the cumulative dose of cyclophosphamide administered was 18,000 mg (about 6 month continuous therapy) (OR 11.9, 95% CI 2.17–65.04). Prednisone also showed a significantly elevated odds ratio when patients received more than 5,000 mg (approximate cumulative dose in six courses) (OR 5.1, 95% CI 1.1–31.3) compared to those who received less than 5,000 mg (OR 1.5, 95% CI 0.3–6.4). Similarly, those who received 10 mg or less of vincristine had a lower risk of second myeloid leukaemia (OR 3.0, 95% CI 0.4–25.5) compared to those who received more (OR 6.2, 95% CI 0.7– 59.7). A test for trend of increasing cumulative dose was significant for cyclophosphamide ($P = < 0.001$) and for prednisone ($P = 0.01$), but was not significant for vincristine ($P = 0.114$).

An increasing risk with increasing average daily doses of drug (mg $day^{-1}$) was observed only with cyclophosphamide. Patients who received more than an average of 37 mg $day^{-1}$ of cyclophosphamide were at a higher risk of developing second myeloid leukaemia (OR 21.6, 95% CI 2.80–166.4) than those who received an average of less than 37 mg $day^{-1}$ (OR 2.8, 95% CI 0.7–11.7).

### Discussion

As in previous studies (Haas *et al.*, 1987; Curtis *et al.*, 1984; Einhorn, 1978; Boivin *et al.*, 1986; Portugal *et al.*, 1979; Rosner *et al.*, 1978; Tucker *et al.*, 1987; Reimer *et al.*, 1977; Kaldor *et al.*, 1990*a*; Kaldor *et al.*, 1990*b*) we observed that chemotherapy for cancer, particularly the administration of cyclophosphamide, is an important risk factor in the development of a subsequent myeloid leukaemia. An elevated risk of myeloid leukaemia due to radiotherapy was not seen and is in agreement with previous reports (Boivin & Hutchison, 1981; Valagussa *et al.*, 1986; Kaldor *et al.*, 1990*a*; Kaldor *et al.*, 1990*b*). This investigation has the advantage of being population-based, and thus is minimally influenced by diagnostic staging, or treatment practices, or by patient characteristics or referral patterns which may be peculiar to single institutions.

The absence of an elevated risk of myeloid leukaemia due to radiotherapy of the first primary is consistent with the analyses of the risk of second primary leukaemia of any type following initial primary Hodgkin's disease (Boivin & Hutchison, 1981; Valagussa *et al.*, 1986; Kaldor *et al.*, 1990*a*) ovarian cancer (Mehnert *et al.*, 1986; Kaldor *et al.*, 1990*b*; Greene *et al.*, 1986) or breast cancer (Mehnert *et al.*, 1986). As in earlier reports (Mehnert *et al.*, 1986), a slight but statistically nonsignificant elevated risk was noted when chronic myeloid leukaemia alone was considered. A larger sample size would be necessary to determine more precisely the effect of radiotherapy in the induction of chronic myeloid leukaemia.

The role of cyclophosphamide as a leukaemogen in acute myeloid leukaemia is consistent with other reports (Haas *et al.*, 1987; Portugal *et al.*, 1979; Kaldor *et al.*, 1990*a*; Kaldor *et al.*, 1990*b*; Greene *et al.*, 1986). Further, there was a suggestion in these data of an increase in risk with increasing dose of cyclophosphamide. In a previous study (Portugal *et al.*, 1979) of breast cancer as the first primary, the median cumulative dose of cyclophosphamide administered was 54,150 mg in 37.5 months. In another report (Greene *et al.*, 1983), exposed cases with non-Hodgkin's lymphoma who developed acute nonlymphatic leukaemia received an average of three times more cyclophosphamide than exposed controls (71,700 mg in cases *vs* 23,300 mg in controls). Kaldor *et al.* (1990*a*) report greatly increased risks of acute or non-lymphocytic leukaemia associated with treatment regimens for Hodgkin's disease containing cyclophosphamide when more than six cycles are administered relative to six or fewer cycles. Similar results are apparent regarding an increase in risk with increase in cyclophosphamide dose in their comparison study of treatment for ovarian cancer (Kaldor *et al.*, 1990*b*). Our results suggest that patients receiving higher doses of cyclophosphamide (intake for more than 5 months or more than 37 mg per day) are at 10 to 20-fold risk of myeloid leukaemia compared to those who did not receive this drug. Due to the relatively small size of this study, however, the degree to which the risks associated with increasing cumulative months and average daily intake are independent could not be adequately evaluated in our data.

An unexpected finding of this study is the increased risk of second primary myeloid leukaemia associated with prior chemotherapy which included prednisone. Specifically, the data indicate that the relative risk of second primary myeloid leukaemia is many times greater when the chemotherapy administered for an initial primary cancer includeds prednisone than when the chemotherapy regimen does not include prednisone. It was possible to demonstrate that the risk of myeloid leukaemia associated with the use of cyclophosphamide is enhanced by prednisone. The risk was even greater when prednisone was administered with chemotherapy drugs other than cyclophosphamide. There was no significantly elevated risk of myeloid leukaemia in patients who were administered these other drugs (except cyclophosphamide) without prednisone. Further analysis of available data showed that patients administered vincristine and 5-fluorouracil with prednisone had significantly elevated risk of second myeloid leukaemia compared to those who received these drugs without prednisone. It remains to be determined whether the cell cycle specific action of these drugs (vincristine at mitotic and S phase; 5-fluorouracil at S phase) has any importance in this context (Carter & Livingston, 1982). Since no patient received prednisone as a single chemotherapy agent, the carcinogenic potential of prednisone alone could not be assessed.

The apparent action of prednisone could not be explained on the basis of being administered more often to patients receiving higher doses of the other chemotherapy agents (either cumulative or average daily dose), nor could it be explained on the basis of use in combination with other drugs known to be carcinogenic in humans (melphalan) or animals (cisplatin and adriamycin). Similarly, no site or sites of first primary cancer were associated with a more common use of prednisone, nor were the uses of radiotherapy and/or hormone therapy associated with the administration of prednisone.

Reviews regarding the carcinogenicity of prednisone have been inconclusive due to a paucity of relevant data from animal and human studies (IARC Monog, 1981). Although its mechanism of action against the cancer cell is unknown (Salmon & Sartorelli, 1987), the role of prednisone as a potential or facilitory carcinogen appears plausible if one considers the available information regarding some of its known actions. A primary action of the glucocorticoids (including prednisone) appears to be the suppression of immunologic activities of the host organism. Cells of lymphoid origin in culture appear to be more specifically inhibited by the glucocorticoids than do other cell lines (Calabresi & Parks, 1985; Wheeler, 1982). Pharmacologic doses of these steroids produce a profound transient lymphopenia in man (Beardsley & Cohen, 1978) and, possibly because of this, prednisone has been particularly effective in bringing early remission in patients with acute lymphatic leukaemia (Blum *et al.*, 1982). This implies that the mechanism of action of prednisone in inducing myeloid leukaemia may be indirect, in that it possibly interferes with certain natural immunologic defence mechanisms, perhaps making the myeloid cell more susceptible or hyperresponsive to other influences such as alkylating agents or other chemotherapy drugs.

Indirect evidence supporting a role of prednisone in leukaemogenesis comes from its presence in the MOPP (Mechlorethamine, Oncovin (vincristine), Prednisone and Procarbazine) combination chemotherapy regimen commonly given in treating Hodgkin's disease patients. Treatment with MOPP has been observed in several studies to be associated with a high risk of subsequent acute nonlymphatic leukaemia compared to the alternative regimen ABVD (Adriamycin, Bleomycin, Vinblastine and Dacarbazine) (Boivin & Hutchison, 1981; Valagussa *et al.*, 1986; Van der Velden *et al.*, 1988; Valagussa *et al.*, 1982). In a recent report (Van der Velden *et al.*, 1988), it was observed that all 18 cases of second primary acute nonlymphatic leukaemia in the study had received vincristine and procarbazine, while about half of the controls received that combination. Although cases in that study were also more likely than controls to have received prednisone in conjunction with other chemotherapy drugs (89% of cases *vs* 52% controls), no analyses were performed to attempt to delineate any difference in risk between those who received and did not receive this steroid. Other studies in which all individual drugs of chemotherapy regimens have been specified show that most of the patients who developed acute nonlymphatic leukaemia had been given prednisone as part of the protocol for treatment of the first primary cancer (Kushner *et al.*, 1988; Greene *et al.*, 1983). The recent findings of Kaldor *et al.*, (1990*a*, 1990*b*) are also generally consistent with this possibility, although MOPP regimens are not analysed separately in their study, but rather are included within a class of treatment characterised by combinations containing nitrogen mustard and procarbazine.

Findings from Stanford also generally support the present results, even though prednisone has been omitted from two cycles of the MOPP treatment regimen for some patients in their series (Tucker *et al.*, 1988). Whether the risks of subsequent leukaemia associated with this modified MOPP therapy are lower than those reported from other centres which always include prednisone in MOPP cannot be adequately evaluated from the published literature.

The single unique finding of this study is the possible role of prednisone as a co-carcinogen in enhancing the leukaemogenic action of other chemotherapy drugs. Although this investigation constitutes the largest sample yet reported for a specific type of leukaemia, a larger number of cases is needed to better evaluate the etiologic role of individual chemotherapeutic agents, particularly in combination with prednisone. Thus, the present results call for more rigorous evaluation of the role of this commonly used steroid in the etiology of leukaemia. In conducting further investigations of second primary leukaemias it will be important to evaluate the use of prednisone in combination chemotherapy, and to consider its intake for other ailments. Research at the experimental and molecular levels may also provide clues regarding drug interaction and carcinogenesis. Detailed information on past and present history of prednisone administration for associated nonmalignant conditions will be of importance in assessing its potential carcinogenic activity.

Thanks are due to Dr M. Krishna Bhargava, Director, Kidwai Memorial Institute of Oncology, Bangalore, India for having deputed Dr A. Nandakumar from his post to take the MPH Program at the University of Washington.

We are indebted to the staff of the Cancer Surveillance System at the Fred Hutchinson Cancer Research Center for access to cancer registry files which helped to initiate this study. We wish to express gratitude to the hospitals and medical record personnel (in King, Pierce and Snohomish counties) for their cooperation in retrieving the medical records of patients for review.

The use of Army medical records (Madigan Army Medical Center, Tacoma) in the preparation of this material is acknowledged, but it is not to be construed as implying official Department of the Army approval of the conclusions presented. Thanks are also due to the physician oncologists who were contacted for obtaining details of chemotherapy from their private clinic files.

This work was supported in part by the following grants from the National Cancer Institute: CA 18221, CA 18029, CA 15704, CA 09515, CA 47658 and CA 01374.

### References

BEARDSLEY, G.P. & COHEN, H.J. (1978). Corticosteroid induced lymphocytopenia in man: absence of splenic influence and effect of recipient serum. *Am. J. Hematol.*, **4**, 255.

BENNETT, J.M. (1986). Classification of the myelodysplastic syndromes. *Clin. Haematol.*, **15**, 909.

BLUM, R.H., FREI, E. & HOLLAND, J.F. (1982). Principles of Dose, Schedule and Combination Chemotherapy. In *Cancer Medicine*. Holland, J.F. & Frei, E. (eds). pp. 730–752. Lea and Febiger: Philadelphia.

BOICE, J.D., BLETTNER, M., KLEINERMAN, R.A. & 35 others (1987). Radiation dose and leukaemia risk in patients treated for cancer of the cervix. *JNCI*, **79**, 1295.

BOIVIN, J.F. & HUTCHISON, G.B. (1981). Leukemia and other cancers after radiotherapy and chemotherapy for Hodgkin's disease. *JNCI*, **67**, 751.

BOIVIN, J.F., HUTCHISON, G.B. & EVANS, F.B. (1986). Leukemia after radiotherapy for first primary cancers of various anatomic sites. *Am. J. Epidemiol.*, **123**, 993.

BRESLOW, N. & DAY, N.E. (1980). Statistical Methods in Cancer Research. *IARC Scientific Publications*, Lyon, **32**, 248.

CALABRESI, P. & PARKS, R.E. Jr (1985). Chemotherapy of neoplastic diseases. In *Goodman and Gilman's, The Pharmacological Basis of Therapeutics*, 7th Edition. pp. 1240–1306. Macmillan Publishing Company: New York.

CARTER, S.K. & LIVINGSTON, R.B. (1982). Drugs available to treat cancer. In *Principles of Cancer Treatment*. Carter, S.K., Glatstein, E. & Livingston, B. (eds). pp. 111–145. McGraw Hill: New York.

CURTIS, R.E., HANKEY, B.F., MYERS, M.H. & YOUNG, J.L. Jr (1984). Risk of leukemia associated with the first course of cancer treatment: an analysis of the Surveillance, Epidemiology and End Results program experience. *JNCI*, **72**, 531.

EINHORN, M. (1978). Acute leukemia after chemotherapy (melphalan). *Cancer*, **41**, 444.

GREENE, M.H., BOICE, J.D. Jr, GREER, B.E., BLESSING, J.A. & DEMPO, A.J. (1982). Acute nonlymphatic leukemia after therapy with alkylating agents for ovarian cancer: a study of five randomized clinical trials. *N. Engl. J. Med.*, **307**, 1416.

GREENE, M.H., YOUNG, R.C., MERILL, J.M. & DEVITA, V.T. (1983). Evidence of a treatment dose response in acute nonlymphocytic leukemias which occur after therapy of non-Hodgkin's lymphoma. *Cancer Res.*, **43**, 1891.

GREENE, M.H., HARRIS, E.L., GERSHENSON, D.M. & 6 others (1986). Melphalan may be a more potent leukemogen than cyclophosphamide. *Ann. Intern. Med.*, **105**, 360.

HAAS, J.F., KITTLEMANN, B., MEHNERT, W.H. & 4 others (1987). Risk of leukemia in ovarian tumor and breast cancer patients following treatment by cyclophosphamide. *Br. J. Cancer.*, **55**, 213.

IARC MONOGRAPHS ON THE EVALUATION OF THE CARCINOGENIC RISK OF CHEMICALS TO HUMANS: SOME ANTI NEOPLASTIC AND IMMUNOSUPPRESSIVE AGENTS (1981). *IARC Scientific Publications*: Lyon, **26**, 305.

INTERNATIONAL CLASSIFICATION OF DISEASES FOR ONCOLOGY (1976). World Health Organization: Geneva. **43**, 45.

JACOBS, A. (1987). Human pre-leukemia: do we have a model? *Br. J. Cancer*, **55**, 1.

KALDOR, J.M., DAY, N.E., CLARKE, A. & 27 others (1990*a*). Leukemia Following Hodgkin's disease. *N. Engl. J. Med.*, **322**, 7.

KALDOR, J.M., DAY, N.E., PETTERSSON, F. & 21 others (1990*b*). Leukemia Following Chemotherapy For Ovarian Cancer. *N. Engl. J. Med.*, **322**, 1.

KOEFFLER, H.P. (1986). Myelodysplastic syndromes (preleukemia). *Seminars in Hematol.*, **23**, 284.

KUSHNER, B.H., ZAUBER, A. & TAN, C.T.C. (1988). Second malignancies after childhood Hodgkin's disease. *Cancer*, **62**, 1364.

MEHNERT, W.H., HAAS, J.F., KITTELMANN, B. & 4 others (1986). A case control study of leukemia as a second primary neoplasm in carcinogenicitiy of alkylating cytostatic drugs. *IARCS Scientific Publications*: Lyon, **78**, 203.

PENN, I. (1982). Second neoplasms following radiotherapy or chemotherapy for cancer. *Am. J. Clin. Oncol.*, **5**, 83.

PORTUGAL, M.A., FALKSON, H.C., STEVENS, K. & FALKSON, G. (1979). Acute leukemia as a complication of long term treatment of advanced breast cancer. *Cancer Treat Rep.*, **63**, 177.

REIMER, R.R., HOOVER, R., FRAUMENI, J.F. & YOUNG, R.C. (1977). Acute leukemia after alkylating agent therapy of ovarian cancer. *N. Engl. J. Med.*, **297**, 177.

ROSNER, F., CAREY, R.W. & ZARRABI, M.H. (1978). Breast cancer and acute leukemia: report of 24 cases and review of the literature. *Am. J. Hematol.*, **4**, 151.

ROSNER, F., GRUNWALD, H.W. & ZARRABI, M.H. (1982). Cancer after the use of alkylating and non-alkylating cytotoxic agents in man. *Cancer Surv.*, **1**, 599.

SALMON, S.E. & SARTORELLI, A.C. (1987). Cancer chemotherapy. In *Basic and Clinical Pharmacology*, 3rd Edition. Katzung, B.G. (ed.). pp. 665–701. Appleton and Lange: Norwalk, Connectitcut.

TUCKER, M.A., MEADOWS, A.T., BOICE, J.D. Jr & 7 others (1978). Leukemia after therapy with alkylating agents for childhood cancer. *JNCI*, **78**, 459.

TUCKER, M.A., COLEMAN, C.N., COX, R.S., VARGHESE, A. & ROSENBERG, S.A. (1988). Risk of second cancers after treatment for Hodgkin's disease. *N. Engl. J. Med.*, **318**, 76.

VALAGUSSA, P., SANTORO, A., FOSSATI BELLANI, F., FRANCHI, F., BANFI, A. & BONADONNA, G. (1982). Absence of treatment induced second neoplasms after ABVD in Hodgkin's disease. *Blood*, **59**, 488.

VALAGUSSA, P., SANTORO, A., FOSSATI BELLANI, F, BANFI, A. & BONDADONNA, G. (1986). Second leukemia and other malignancies following treatment for Hodgkin's disease. *J. Clin. Oncol.*, **4**, 830.

VAN DER VELDEN, J.W., VAN PUTTEN, W.L.J., GUINEE, V.F. & others (1988). Subsequent development of acute non-lymphocytic leukemia in patients treated for Hodgkin's disease. *Int. J. Cancer.*, **42**, 252.

WHEELER, G.P. (1982). Alkylating agents. In *Cancer Medicine*. Holland, J.F. & Frei, E. (eds). pp. 824–843. Lea and Febiger: Philadelphia.

WOLF, B.C. & NEIMAN, R.S. (1988). The bone marrow in myeloproliferative and dysmyelopoietic syndromes. *Hematol/Oncol. Clinics of N. America*, **2**, 669.